# Alternative catalytic residues in the active site of Esco acetyltransferases

**DOI:** 10.1038/s41598-020-66795-z

**Published:** 2020-06-17

**Authors:** Tahereh Ajam, Inessa De, Nikolai Petkau, Gabriela Whelan, Vladimir Pena, Gregor Eichele

**Affiliations:** 1 Genes and Behavior Department, Max Planck Institute for Biophysical Chemistry, 37077 Göttingen, Germany; 2 Research Group Macromolecular Crystallography, Max Planck Institute for Biophysical Chemistry, 37077 Göttingen, Germany; 3 Present Address: European Molecular Biology Laboratory (EMBL), Structural and Computational Biology Unit, Meyerhofstrasse 1, 69117 Heidelberg, Germany; 4 Present Address: Structural Biology Division, The Institute of Cancer Research, SW3 6JB London, United Kingdom

**Keywords:** Enzyme mechanisms, Cohesion, X-ray crystallography

## Abstract

Cohesin is a protein complex whose core subunits, Smc1, Smc3, Scc1, and SA1/SA2 form a ring-like structure encircling the DNA. Cohesins play a key role in the expression, repair, and segregation of eukaryotic genomes. Following a catalytic mechanism that is insufficiently understood, Esco1 and Esco2 acetyltransferases acetylate the cohesin subunit Smc3, thereby inducing stabilization of cohesin on DNA. As a prerequisite for structure-guided investigation of enzymatic activity, we determine here the crystal structure of the mouse Esco2/CoA complex at 1.8 Å resolution. We reconstitute cohesin as tri- or tetrameric assemblies and use those as physiologically-relevant substrates for enzymatic assays *in vitro*. Furthermore, we employ cell-based complementation studies in mouse embryonic fibroblast deficient for Esco1 and Esco2, as a means to identify catalytically-important residues *in vivo*. These analyses demonstrate that D567/S566 and E491/S527, located on opposite sides of the murine Esco2 active site cleft, are critical for catalysis. Our experiments support a catalytic mechanism of acetylation where residues D567 and E491 are general bases that deprotonate the ε-amino group of lysine substrate, also involving two nearby serine residues - S566 and S527- that possess a proton relay function.

## Introduction

Cohesin is a ring-like protein complex that is composed of the four subunits Smc1, Smc3, Scc1 (kleisin), and SA1/SA2. Smc1 and Smc3 belong to the family of SMC (structural maintenance of chromosomes) proteins, and have ATPase head domains. Cohesin mediates sister chromatid cohesion, following a mechanism that is still under investigation. In one model, Chapard *et al*. recently proposed that sister chromatids are entrapped in a ring compartment formed by Scc1 and the ATPase domains of Smc1 and Smc3^1^. Xu and Yanagida propose a model in which DNA is captured in a ring compartment formed by an interaction of the hinge and head domains of Smc1 and Smc3^2^. In addition to sister chromatid cohesion, cohesin facilitates postreplicative homologous recombination repair of double-strand DNA breaks, and regulates gene expression *via* the formation of chromatin loops^3–7^. Cohesin is loaded onto chromatin by the Scc2-Scc4 loader complex and released by Wapl and Pds5^8–13^. Both cohesin loading and unloading depend on the ATPase activity of the Smc head domains^8,14–17^.

Recent studies suggest that once cohesin is loaded onto chromatin, DNA interacts with a basic patch residing on the Smc3 head domain and thereby stimulates its ATPase activity. A notable structural feature of this basic patch is the presence of two neighboring conserved lysines^17,18^. Acetylation of these residues by yeast acetyltransferase Eco1 or its mammalian orthologues Esco1 and Esco2 (establishment of cohesion) decreases the positive charge of the patch, which weakens DNA binding and lessens ATPase activity^17^. This in turn counteracts the activity of the release factors Wapl-Pds5. As a result, Esco activity stabilizes cohesin on DNA^19^. In vertebrates, cohesion establishment additionally involves Sororin, which competes with Wapl for binding to Pds5 and in this way counteracts the releasing activity of Wapl-Pds5^20,21^.

Esco1 and Esco2 belong to the GNAT (GCN5-related N-acetyltransferase) family. These two isozymes consist of divergent N‐termini followed by a C2H2 zinc finger and a conserved C-terminal acetyltransferase domain^22^. Esco1 and Esco2 differ in several respects. Esco1 is evenly expressed throughout the cell cycle, while Esco2 is highly abundant during the S-phase^23,24^. Esco1 but not Esco2 interacts directly with cohesin *via* Pds5^25^. Esco2 interacts with the replication proteins, PCNA (proliferating cell nuclear antigen)^26,27^ and MCM (minichromosome maintenance protein complex)^28,29^. Esco1 mutation is associated with endometrial cancer^30^ and mutations in Esco2 cause RBS (Roberts syndrome), a congenital disease^31–33^. In RBS, metaphase chromosomes show a loss of cohesion in the pericentric heterochromatin while cohesion is maintained in the arms^34^. A significant fraction of *Esco1*-deficient mice is viable (this study), while *Esco2*-deficient mice always die early in development^24^.

An acetyltransferase typically employs a general base to deprotonate the ε-amino group of a substrate lysine which initiates acetyl transfer from AcCoA. Intriguingly, crystal structure of human acetyltransferase HsESCO1, reported by Kouznetsova *et al*., led the authors to propose that the general base is provided by the substrate rather than the enzyme. Thus, D107 of SMC3 might deprotonate K105 and K106 of SMC3^35^. In contrast, Rivera-Colon *et al*. proposed that D810 of HsESCO1 plays the role of a general base. Indeed, mutation of this aspartate to asparagine abrogates acetylation of an Smc3 peptide mimicking SMC substrate^36^. A subsequent study by Chao *et al*. has observed that in the *Xenopus* xEco2/Smc3 peptide structure, the Smc3 D107 does not point towards the ϵ-amino group of the substrate lysines but interacts with two conserved R621 and W623 residues of xEco2. This suggests that D107 of Smc3 plays a role tethering the enzyme to the substrate rather than acting as a general base^37^. In agreement with Rivera-Colon *et al*., Chao *et al*. propose that D677 (the equivalent of D810 of HsESCO1) could serve as a general base. However, both studies noted that this particular aspartic acid is not strictly conserved among Esco homologs, suggesting that other residues at the active site may also contribute to catalysis.

To shed light into the catalytic mechanism of Esco1 and Esco2, we determined the crystal structure of murine Esco2 in complex with CoA at atomic resolution, established a robust framework for assessing enzymatic activity *in vitro* and *in vivo*, and performed mutagenic analysis of the active center. An alternative catalytic model is proposed.

## Results

### Structure of MmEsco2^368–592^ in complex with Coenzyme A

**Figure 1:**
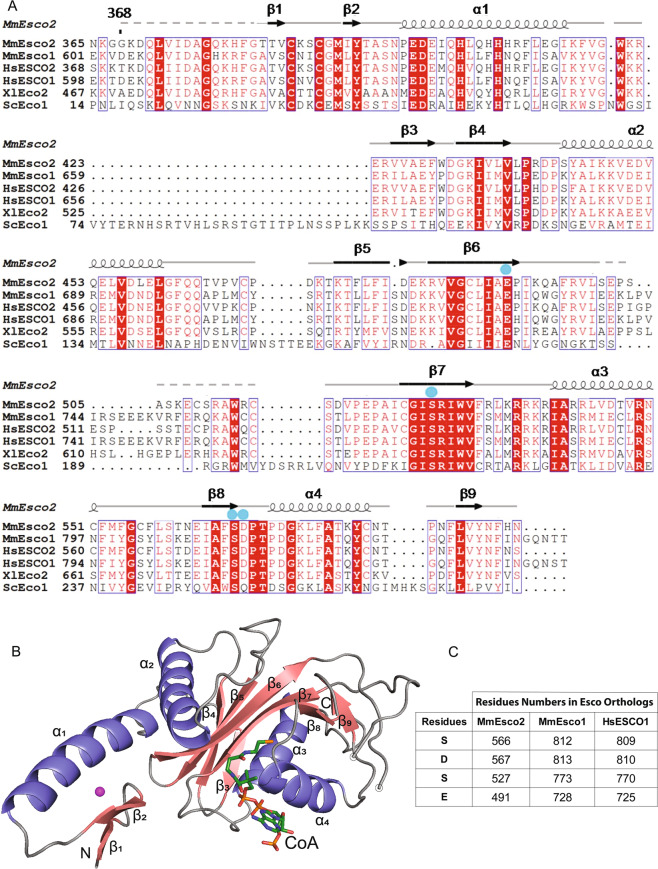
Structure of the MmEsco2^368–592^/CoA complex. (**A**) Sequence alignment^49^ of Esco orthologs. Sequences shown are *Mus musculus* MmEsco1, MmEsco2, *Homo sapiens* HsESCO1, HsESCO2, *Xenopus laevis* XlEco2 and *S. cerevisiae* ScEco1. Invariant residues are shown with a red background, and highly conserved residues are boxed. Numbering and secondary structural elements above the sequence alignment are for the MmEsco2^368–592^ sequence. Dashed lines mark the disordered regions. Blue circles indicate residues that might be involved in the abstraction of the proton from the ε-amino group of the substrate lysine. (**B**) Ribbon representation of the MmEsco2^368–592^/CoA complex. α-helices are shown in blue, β-strands in raspberry, and loop regions in grey. CoA is represented as sticks and colored according to elements: carbon, green; nitrogen, blue; sulfur, orange; oxygen, red and the zinc ion shown as a magenta sphere. There is an unresolved region in a loop connecting β6 and β7. Start and end point of this region is indicated by empty circles. (**C**) Numbering of equivalent putative catalytic residues of MmEsco2 in MmEsco1 and HsESCO1 sequences. Figure adapted from^50^.

We crystallized MmEsco2^368–592^ protein consisting of the C2H2 zinc finger and the acetyltransferase domain. Crystals diffracted to 1.8 Å resolution. By making use of the natively bound zinc ion, the crystal structure was determined by SAD (single-wavelength anomalous dispersion) from a dataset collected at the zinc peak wavelength (Supplementary Table S1). The refined MmEsco2^368–592^ structure revealed continuous electron density, except for two short, structurally disordered and hence unresolved regions (residues 368–383 and 501–514, Fig. 1A). The zinc finger (residues 385–416), which is located N-terminally of the catalytic domain, consists of two β-strands and one α-helix that encircle the zinc ion (Fig. 1B). Residues 423–592 of MmEsco2^368–592^ reveal an overall fold similar to that of other GNAT family acetyltransferases^38^. This fold consists of a conserved catalytic domain (β5, β6, β7, α3, and α4) flanked by structurally variable regions (Fig. 1B). The CoA cofactor is natively present in a complex with MmEsco2 in a groove formed by β7 and β8 strands and α3 and α4 helices (Fig. 1B). This is reminiscent of the position of the CoA or AcCoA in other GNAT family members^38^.

### The active site architecture of MmEsco2^368–592^ and identification of candidate catalytic residues

**Figure 2:**
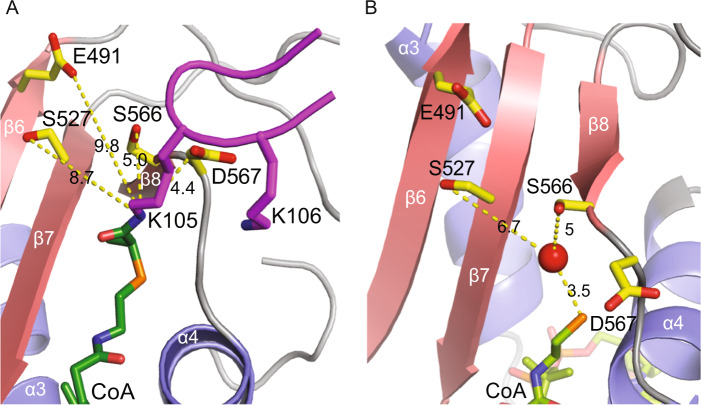
The active site of the MmEsco2^368–592^/CoA complex. (**A**) Close-up view of the active site of the MmEsco2^368–592^/K105-CoA model. The position of the K105 was modeled by superposition with the structure of xEco2 in complex with a K105-CoA peptide [PDB ID code 5N1W]. Side chains of putative catalytic residues are shown. Dashed lines indicate the distances in angstrom from relevant atoms of the putative catalytic residues to the ε-amino group of K105. Smc3 peptide is shown in magenta. (**B**) A water molecule, shown as a red sphere, is located in the active site of MmEsco2^368–592^/CoA between the candidate catalytic residues and CoA. Dashed lines indicate the distances in angstrom from relevant atoms of the putative catalytic residues to the water molecule.

We searched for residues in the active site cleft of MmEsco2^368–592^, which could act as a general base for catalyzing the nucleophilic attack of the lysine ε-amino group on the AcCoA thioester bond. Structural superposition of MmEsco2^368–592^ with xEco2 in complex with a Smc3 peptide conjugated with CoA at K105^37^ enabled identification of candidate catalytic residues in MmEsco2. The most obvious candidate residue is D567 that may act in conjunction with S566; the latter potentially acting as a proton relay. It is noteworthy that the equivalent D810 was previously suggested as general base in HsESCO1^36^ (for an overview of residues equivalence, see Fig. 1C). S566 and D567 are located at the CoA binding pocket, S566 in the C-terminus of the β8 strand and D567 in a flexible loop connecting β8 strand and α4 helix (Fig. 2A). The γ-oxygen of S566 and δ-oxygen of D567 are ~ 5 and ~ 4.4 Å away from the ε-amino group of K105 (Fig. 2A and Supplementary Fig. S2A). The distance of the δ-oxygen of D567 and the γ-oxygen of S566 is 5.7 Å, consistent with a proton relay function. We also considered S527 as a possible relay, with its γ-oxygen being ~ 8.7 Å away from ε-amino group of K105 (Fig. 2A and Supplementary Fig. S2A). The ε-oxygen of E491 is ~ 9.8 Å away from ε-amino group of K105 (Fig. 2A and Supplementary Fig. S2A) and thus E491 could also serve as general base. We also fitted MmEsco2^368–592^ to xEco2 in complex with a Smc3 peptide conjugated with CoA at K106. Based on this comparison, we arrived at the same candidate catalytic residues as for K105 (Supplementary Fig. S2A).

Notably, a water molecule located between these four residues and CoA, similar to the one observed in the xEco2/K106-CoA structure^37^, might be involved in proton transfer (Fig. 2B). The residues E491, S527 and S566 are invariant among Esco homologs. D567 residue is also highly conserved, except for yeast Eco1 where this residue is a glutamine (Fig. 1A).

### Establishing of an acetylation assay that mimic the physiological conditions

To investigate whether the selected candidate residues contribute to catalysis, we mutated S566 and/or D567 and measured catalytic activities with trimeric or tetrameric cohesin ring substrates. Expression of full-length MmEsco1 and MmEsco2 wild type proteins gave very low yields, but recombinant full-length HsESCO1 was readily expressed in Sf9 cells (Supplementary Fig. S1A). Of note, the structure of the active site of MmEsco2 and HsESCO1 are very similar (Supplementary Fig. S2B). Thus, we applied HsESCO1 for the following reconstitution-based assays.

A major drawback of the enzymatic assays reported before for Esco proteins is that either autoacetylation or acetylation of peptide mimetics were used, rather than physiological substrates. Recombinant cohesin assembled as a trimer was also employed, although it does not thoroughly represent physiological substrate^39^. To enhance the relevance and accuracy of our observations, we therefore decided to reconstitute entire cohesin rings recombinantly and use them for enzymatic assays.

**Figure 3:**
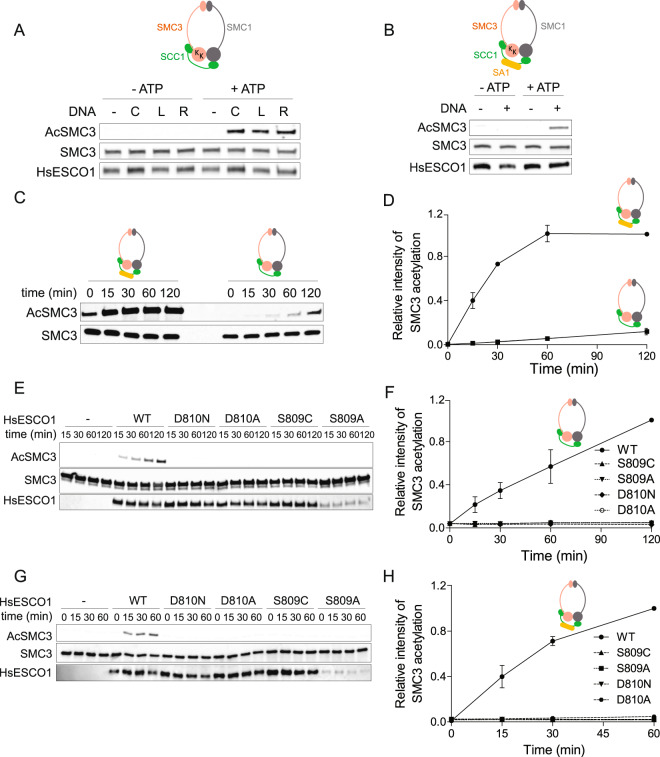
S809 and D810 are required for HsESCO1 activity to acetylate recombinant cohesin substrates. (**A**) The purified recombinant trimeric cohesin complex was incubated with HsESCO1 and AcCoA in the presence or absence of ATP and different topologies of the pcDNA3 plasmid (linear DNA [L], relaxed circular DNA [R] or covalently closed circular DNA [C]). (**B**) The purified tetrameric cohesin complex was incubated with HsESCO1 and AcCoA in the presence or absence of ATP and covalently closed circular DNA. (**C**,**D**) Time course of SMC3 acetylation after incubation of purified trimeric or tetrameric cohesin complexes with HsESCO1 in the presence of ATP, DNA and AcCoA. Half-times for the tetramer is ~ 20 min. Acetylation of the trimer is considerably slower. (**E**,**F**) Time course of SMC3 acetylation after incubation of purified trimeric cohesin with wild-type or mutants of HsESCO1. (**G**,**H**) Time course of SMC3 acetylation after incubation of purified tetrameric cohesin with wild-type or mutants of HsESCO1. All data were normalized to maximum signal and are shown as mean ± SEM (n = 2). Figure adapted from^50^.

We first generated human trimeric (Supplementary Fig. S1B) and tetrameric recombinant cohesin (Supplementary Fig. S1C), as previously described^39^. Trimeric and tetrameric substrates were acetylated by HsESCO1, at K105 and K106 of SMC3 in the presence of ATP, circular, linear or relaxed DNA (Fig. 3A,B). To detect SMC3 acetylation, we used a monoclonal antibody that specifically recognizes Smc3 singly acetylated on K106 or doubly acetylated on K105 and K106^21^. Acetylation was not detected in reconstituted cohesin substrate when ATP hydrolysis was inhibited^39^ (Supplementary Fig. S1D) or DNA was absent (Fig. 3A,B). Importantly, tetrameric substrate was much more efficiently acetylated than trimeric cohesin (Fig. 3C,D). We conclude that in presence of ATP and DNA, HsESCO1 efficiently acetylates cohesin rings. Since DNA stimulates ATPase activity of the cohesin complex^17,18^, which in turn is required for Smc3 acetylation^39^, it is likely that DNA has an indirect effect on Smc3 acetylation.

Given that cohesin rings are efficiently acetylated only in the tetrameric form, and that acetylation is dependent of ATP and DNA, we conclude that the assay that we established recapitulates, to a good extent, the expected occurrence of HsESCO1 acetylation under physiological conditions.

### Mutations in putative catalytic residues affect acetyltransferase activity of Esco1 in reconstituted *in vitro* assay

Having a reconstituted system at hand that mimics physiological conditions, we next quantified the rate of SMC3 acetylation for site-directed mutants of residues S809 and D810. Recombinant mutant proteins HsESCO1^S809A^, HsESCO1^S809C^, HsESCO1^D810A^, and HsESCO1^D810N^ were produced at similar levels as wild-type control, except for HsESCO1^S809A^ which gave a lower yield. Recombinant proteins were incubated with cohesin tri- and tetrameric complexes. Wild-type HsESCO1 efficiently acetylated both substrates (Fig. 3E–H). Remarkably, none of the mutant proteins acetylate cohesin trimers or tetramers (Fig. 3E–H). Rivera-Colon *et al*.^36^ reported that wild-type protein and the four mutants used here show similar thermal stability, arguing against the possibility that mutant HsESCO1 proteins were unfolded and hence became catalytically inactive. Together, these results indicate that D810 and the neighboring S809 are required for catalytic activity of the HsESCO1 enzyme, when reconstituted recombinant cohesin substrates are used. We infer from the HsESCO1 experiments that S566 and D567 of MmEsco2 are catalytic residues.

### Mutations in putative catalytic residues affect acetyltransferase activity of Esco1 and Esco2 in cells

The existing interactions between several accessory proteins and the tetrameric cohesin ring rises the possibility that the former affect the geometry of the active site pocket and the way substrates bind. Therefore, we asked whether the serine and aspartic acid mutants, completely inactive in the above reconstitution assays, are also catalytically inactive when assayed in mouse embryonic fibroblasts (MEFs). These cell-based complementation assays involve transfection of full-length wild-type or mutant *MmEsco1/2* into MEFs lacking either MmEsco1 or MmEsco2.

**Figure 4:**
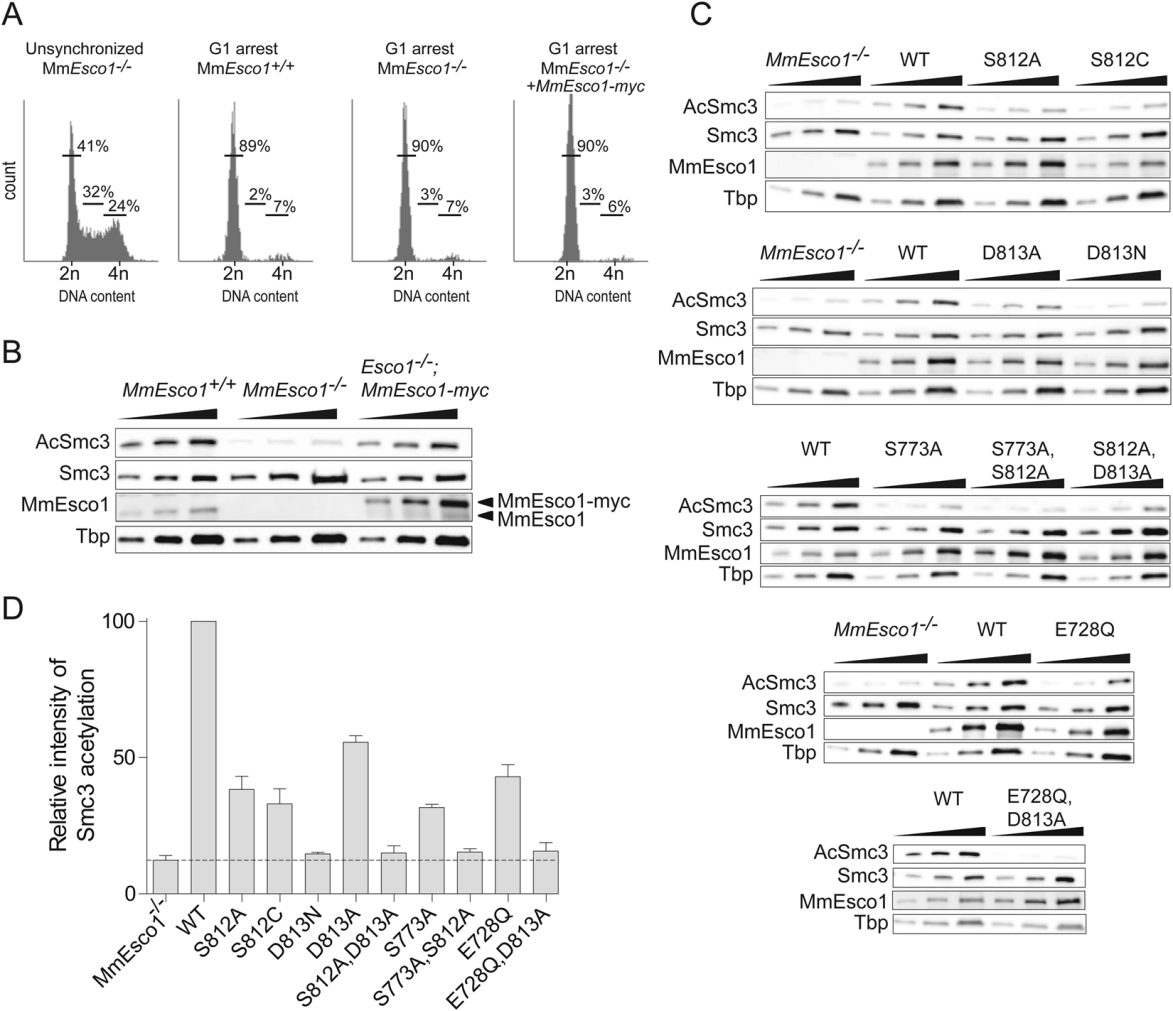
S812, D813, S773, and E728 function cooperatively in the catalysis of MmEsco1. (**A**) Flow cytometry profiles of G1-phase arrested MEFs^*Esco1+/+*^, MEFs^*Esco1−/−*^ and MEFs^*Esco1−/−*^ expressing ectopic wild-type MmEsco1. Unsynchronized MEFs^*Esco1−/−*^ were used as reference. The numbers show the percentage of cells in G1, S, G2/M phase. (**B**) Immunoblot of MEFs^*Esco1+/+*^, MEFs^*Esco1−/−*^ and MEFs^*Esco1−/−*^ expressing ectopic wild-type MmEsco1, arrested in G1-phase. Of note, transiently transfected MmEsco1 was expressed at a level of about 4-fold that of endogenous MmEsco1. (**C**) Immunoblots of MEFs^*Esco1−/−*^ transiently expressing wild-type or mutant versions of MmEsco1, arrested in G1-phase (Supplementary Fig. S4A). Note that MEFs^*Esco1−/−*^ expressed comparable levels of different MmEsco1 variants. (**D**) Quantification of the data shown in C. The dotted line indicates the Smc3 acetylation value for MEFs^*Esco1−/−*^. Data were normalized to wild-type signal and are shown as mean ± SEM (n = 3). In all immunoblotting experiments, the chromatin-bound fractions were analyzed using a 2-fold serial dilution. Figure adapted from^50^.

To obtain *Esco1*-deficient MEFs (MEFs^*Esco1−/−*^), an *Esco1* conditional knockout mouse was generated using the Cre/LoxP system allowing deletion of exons 2 and 3 (Supplementary Fig. S3A–D). MEFs^*Esco1−/−*^ arrested in G1 (Fig. 4A), showed a strong reduction in Smc3 acetylation compared to controls (Fig. 4B). Residual Smc3 acetylation is likely due to MmEsco2 that is present at low-amounts during the G1-phase^23,24^. Transfection of MEFs^*Esco1−/−*^ with *MmEsco1-myc* restored strong Smc3 acetylation (Fig. 4B). Next, we transiently transfected MEFs^*Esco1−/−*^ with single residue mutants that were catalytically inactive in the *in vitro* reconstitution experiments (Fig. 3E–H). It should be noted that all MmEsco1-myc mutants tested were chromatin bound (Supplementary Fig. S4C). Transfection of MEFs^*Esco1−/−*^ with *MmEsco1*^*S812A*^, *MmEsco1*^*S812C*^, and *MmEsco1*^*D813A*^ showed significant acetyltransferase activity, reaching up to 30–50% of wild-type level in contrast to the reconstitution-based assays (Fig. 4C,D). Note that mutant and wild-type cells were equally arrested in G1 (Supplementary Fig. S4A).

Rivera-Colon *et al*. showed that HsESCO1^D813N^ does not acetylate SMC3 peptide substrate^36^, which is consistent with our reconstitution experiments with cohesin ring substrates (Fig. 3F,H). When we transfected MEFs^*Esco1−/−*^ with *MmEsco1*^*D813N*^, Smc3 acetylation was completely abolished (Fig. 4C,D). It is known from the structure of the xEco2/substrate complex that this aspartic acid interacts with the (non-acetylated) substrate lysine via a salt bridge^37^. Thus, the strong effect of D813N mutation is likely caused by the loss of the general base character as well as of the interference of asparagine with the positioning of the ε-amino group of the substrate lysines. The substitution of residues S812 and D813 with alanine (S812A;D813A) has also abolished Smc3 acetylation (Fig. 4C,D). The hydrophobic environment created by the methyl side chains of the two alanine residues probably leads to poor substrate binding and thus the mutated enzyme cannot carry out acetylation.

Of note, single mutant D813A still exhibits considerable activity (Fig. 4C,D), suggesting that E728 (Figs. 1C and 2A) takes over the role of a general base. Consistent with a mechanism that involves a second general base residue is the observation that the double mutant protein E728Q;D813A resulted in base-line level activity (Fig. 4C,D). As the active site geometry places the carboxyl group of E728 almost 10 Å away from the ε-amino group of K105, S812 and S773 might act as proton shuttles. Consistently, we find that S812A;S773A double mutant is nearly inactive (Fig. 4C,D).

**Figure 5:**
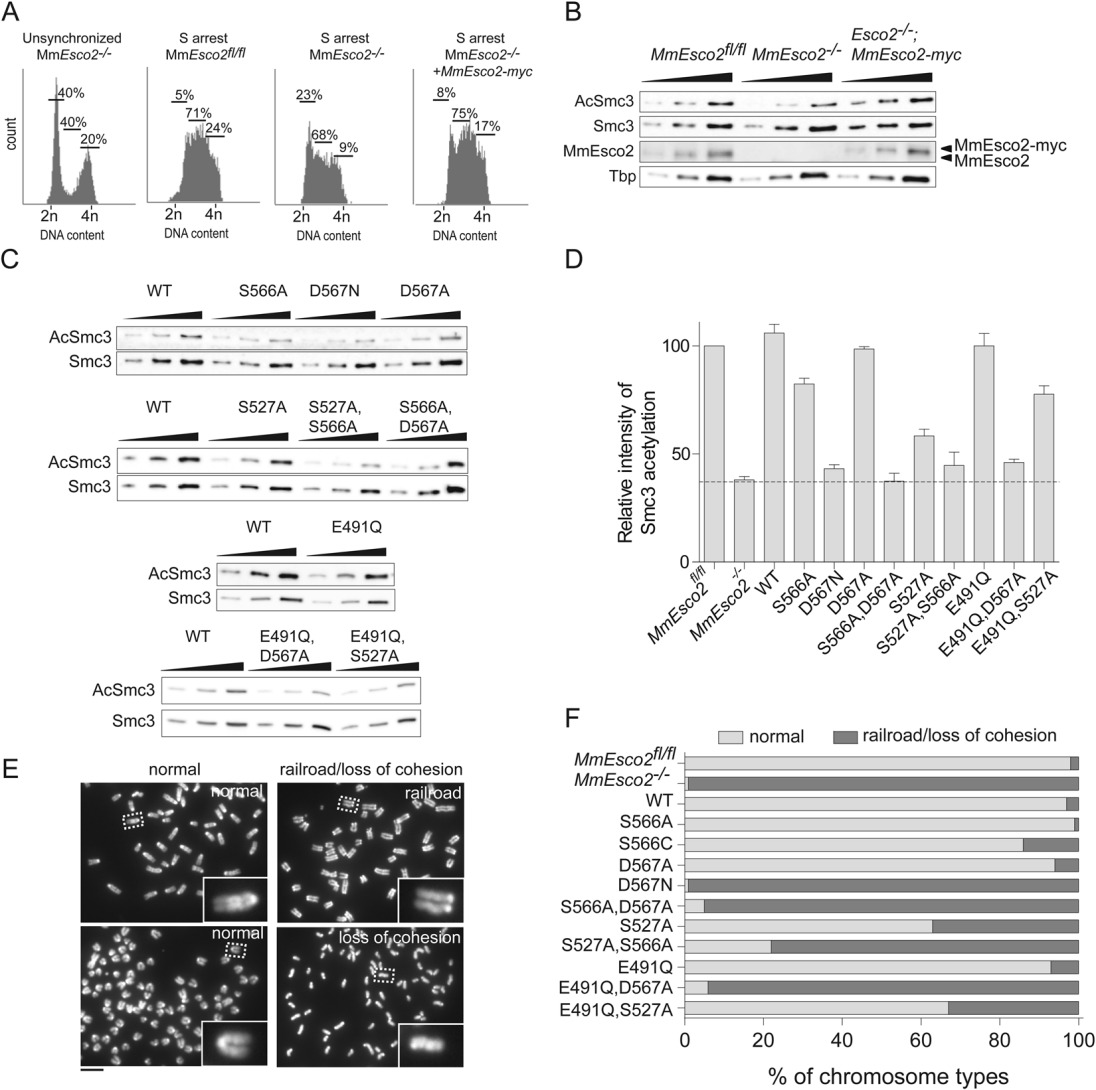
S566, D567, S527, and E491 function cooperatively in the catalysis of MmEsco2. (**A**) Flow cytometry profiles of S-phase arrested MEFs^*Esco2fl/fl*^, MEFs^*Esco2−/−*^ and MEFs^*Esco2−/−*^ expressing ectopic wild-type MmEsco2. Unsynchronized MEFs^*Esco2−/−*^ were used as reference. The numbers show the percentage of cells in G1, S, G2/M phase. (**B**) Immunoblot of MEFs^*Esco2fl/fl*^, MEFs^*Esco2−/−*^ and MEFs^*Esco2−/−*^ expressing ectopic wild-type MmEsco2, arrested in S-phase. Of note, stably transfected MmEsco2 was expressed at a similar level as the endogenous MmEsco2. (**C**) Immunoblot of MEFs^*Esco2−/−*^ stably expressing wild-type or mutant versions of MmEsco2, arrested in S-phase (Supplementary Fig. S4B). Note that MEFs^*Esco2−/−*^ express comparable levels of Esco2 variants (see Supplementary Fig. S4D). (**D**) Quantification of the data shown in C. The dotted line indicates the Smc3 acetylation level seen in MEFs^*Esco2−/−*^. Data were normalized to signal of MEFs^*Esco2fl/fl*^ and are shown as mean ± SEM (n = 3). In all immunoblotting experiments, the chromatin-bound fractions were analyzed using a 2-fold serial dilution. (**E**) Representative prometaphase chromosomes spreads including normal (in MEFs^*Esco2fl/fl*^), railroad and single chromatids (in MEFs^*Esco2−/−*^). Scale bar: 10 µm. (**F**) Frequency of chromosome types were assessed in 1000 prometaphase chromosomes. Figure adapted from^50^.

So far, we have identified several MmEsco1 residues that mediate Smc3 acetylation in cells. We next examined whether the corresponding residues are also involved in catalysis in MmEsco2. Wild-type, *Esco2*^*−/− *^^24^, and *Esco2*^*−/−;Esco2-myc*^ MEFs were arrested in S-phase (Fig. 5A) at which time endogenous MmEsco2 is known to be maximally expressed^23,24^. Consistent with previous published data^24^, MEFs^*Esco2−/−*^ showed a 60% reduction in Smc3 acetylation compared to controls (Fig. 5B,D). Residual Smc3 acetylation is most likely due to MmEsco1, which is expressed throughout the cell cycle^23,24^. Stable transfection of MEFs^*Esco2−/−*^ with *MmEsco2-myc* restored Smc3 acetylation to wild-type levels (Fig. 5B,D). Next, we stably transfected MEFs^*Esco2−/−*^ with corresponding MmEsco2-myc mutants, all of which were chromatin bound (Supplementary Fig. S4D). Reminiscent of the experiments done with MmEsco1 mutants, single mutants *MmEsco2*^*S566A*^, *MmEsco2*^*D567A*^, *MmEsco2*^*S527A*^ and *MmEsco2*^*E491Q*^ were still able to acetylate Smc3 to some extent (Fig. 5C,D). This was not the case with *MmEsco2*^*D567N*^ and the double mutants *MmEsco2*^*S566A;D567A*^, *MmEsco2*^*S527A;S566A*^ and *MmEsco2*^*E491Q;D567A*^ (Fig. 5C,D; for synchronization of mutant cells see Supplementary Fig. S4B). Additionally, we stably transfected MEFs^*Esco2−/−*^ with the double mutant *MmEsco2*^*E491Q;S527A*^. This mutant version acetylates Smc3 at the level of the single mutant S527A (Fig. 5C,D), supporting involvement of the catalytic candidate residues at the other wall of the active site (S566 and D567) in catalysis.

While these results are in close agreement with the effect of the same mutants on MmEsco1 activity, the effect of single mutations (S566A, D567A, S527A and E491Q) on Smc3 acetylation was slightly less pronounced than that observed for MmEsco1. For instance, MmEsco2^S566A^ and MmEsco2^S527A^ showed up to 60–80% residual acetyltransferase activity relative to wild-type (Fig. 5C,D). In the case of Esco1, the residual activity was in the 30–50% range. In addition, MmEsco2^D567A^ and MmEsco2^E491Q^ single mutants acetylated Smc3 to the same extent as its wild-type control (Fig. 5C,D). These differences between Esco1 and Esco2 might be caused by the different chromatin context in which the two enzymes act.

In summary, our data indicate that both MmEsco1 and MmEsco2 engage alternative catalytic residues, supporting a shared mechanism of acetylation despite the distinct biological contexts in which the two enzymes function.

### Effect of catalytic mutants of MmEsco2 on sister chromatid cohesion

RBS patients are characterized by loss of function mutations in HsESCO2 causing sister chromatid cohesion defects^32,33^. Such defects are also seen in *Esco2*-deficient MEFs (Fig. 5E;^24^). We examined which of the MmEsco2 mutants lead to a cohesion defect phenotype. MEFs^*Esco2−/−*^ were stably transfected with wild-type or different mutant versions of *MmEsco2* and synchronized in prometaphase, isolated by mitotic shake-off, and used for chromosome spread preparations. Consistent with the Smc3 acetylation experiments (Fig. 5C,D), chromosome morphology analysis revealed that the single or double mutants MmEsco2^S566A^, MmEsco2^D567A^, MmEsco2^S527A^, MmEsco2^E491Q^ and MmEsco2^E491Q;S527A^ that still enable acetyltransferase activity (Fig. 5C,D) restore wild-type appearance of cohesion to a good extent (Fig. 5F). However, catalytically inactive single or double mutants MmEsco2^D567N^, MmEsco2^S566A;D567A^, MmEsco2^S527A;S566A^ and MmEsco2^E491Q;D567A^, exhibited strong sister chromatid cohesion defects (Fig. 5F). As would be expected, the sister chromatid cohesion assay points to the same catalytic residues as the acetylation assays.

## Discussion

The initial step of a lysine acetyltransferase reaction is deprotonation of the ε-amino group of substrate lysine, mediated by a general base, most commonly an aspartate or a glutamate. Subsequently, the deprotonated ε-amino group carries out a nucleophilic attack on the carbonyl carbon of enzyme-bound AcCoA. This eventually results in the transfer of the acetyl moiety from AcCoA to the substrate lysine.

The active site cleft of Esco acetyltransferases has two walls. One consists of α-helices 3 and 4, whose side chains position AcCoA, and a loop that connects a short β-strand (β8) to α-helix 4. The opposite wall consists of β-strands 5, 6 and 7 (Fig. 1A,B). Candidate catalytic residues protrude from the walls (Fig. 2A). Previous attempts to identify catalytic residues by mutational analyses of Esco1 and Esco2 homologs did not arrive at the same results. In the case of HsESCO1, an aspartate residue belonging to the substrate has been proposed to act as a general base^35^. However, in a more recent study^36^ this role was attributed to an aspartate (D810) from the active site of HsESCO1. Mutation of this highly conserved aspartate to an asparagine inactivated the enzyme, according to an enzymatic assay that made use of Smc3 peptide as a substrate. The crystal structure of xEco2, the Xenopus homolog of Esco1 and Esco2, complexed with an isolated peptide of the Smc3 substrate supports the catalytic role of this aspartate as a general base^37^.

**Figure 6:**
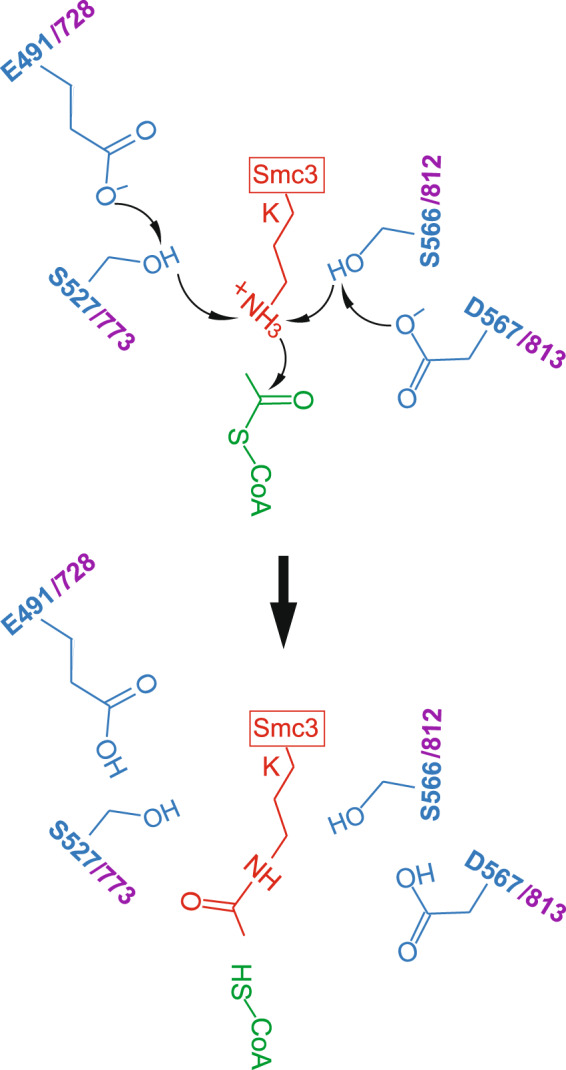
Proposed catalytic mechanism for Esco1 and Esco2. The proposed mechanism involves D567 and/or E491, acting as a general base, to initiate the reaction by abstracting a proton from the hydroxyl group of S566 and/or S527. Subsequently, the hydroxylate of S566 and/or S527 can then act as base catalyst to deprotonate the amino group of K105 and/or K106 (located on a hairpin in Smc3 structure^51^). Subsequently, the nucleophilic attack of the amine on the carbonyl carbon of AcCoA occurs. Black arrows indicate proton transfer. The numbering of putative catalytic residues is based on the MmEsco2 (in blue) and MmEsco1 (in purple) sequences (see Fig. 1C for residue equivalence).

Our own structure-function studies of MmEsco2 also identified this particular aspartate (D567 in MmEsco2, Fig. 6), located on the loop between β8 and α4, as a general base. A D567N mutation was enzymatically inactive in reactions using recombinant trimeric and tetrameric cohesins as substrates. Furthermore, this particular mutant failed to acetylate Smc3 in cells and establish sister chromatid cohesion.

Surprisingly, although mutation of D567 to an alanine abolished acetylation *in vitro*, the enzyme showed appreciable activity *in vivo*, as observed in two different cell-based assays. This indicates that the role of D567 as a general base is taken over by another residue. One possible candidate is E491, located on the β-strand 6 at the opposite of the CoA binding pocket (Fig. 2A). Mutation of the equivalent residue from HsESCO1 to a glutamine was previously shown to abolish acetylation of an SMC3 peptide^36^. Notably, this mutation reduces partially the enzyme activity in cell-based assays, while the double mutant E491Q; D567A is completely inactive in such assays. These observations support the view that both of these residues from the active site cleft act as general bases.

Serine residues, S527 and S566, are located in close proximity to E491 and D567, respectively (Fig. 2A). Mutation of S566 to either a cysteine or an alanine abolishes acetylation *in vitro* (Fig. 3E–H). However, both mutants were partially active in cell-based assays (Fig. 4C,D). Crystal structure of MmEsco2 carrying the S566C mutation is virtually identical with the wild-type protein (data not shown), suggesting that substitution of this residue impairs catalysis rather than the structure. Mutation of the second serine residue, S527A, is known to abolish *in vitro* acetylation of the model peptide^36^. This is consistent with our observation that S527A reduces substantially acetyltransferase activity in cell-based assays (Fig. 4C,D). Taken together, these experiments argue for roles of S527 and S566 in catalysis, possibly acting as a proton relay for E491 and D567 (Fig. 6). A proton relay function of serine has been proposed for the Dat acetyltransferase, in which a glutamate residue deprotonates the substrate amino group through a serine residue^40^. Whether a nearby water molecule (Fig. 2B) is also involved in proton transfer is as yet unclear. In support of this idea is the finding that a water molecule is found at the same position in xEco2^37^.

Our work highlights the crucial importance of designing and employing suitable enzymatic assays, when complex substrates such as cohesin are involved. First, we observed *in vitro* that the presence of SA1 in the composition of tetrameric cohesin (therefore a closer mimic of the physiological context) enables a notably more efficient acetylation than the trimeric version (Fig. 3C,D). Furthermore, mutations that abolish enzymatic activity versus this tetrameric cohesin substrate (e.g. S566A, S566C and D567A) still enable acetylation of cohesin *in vivo*, where even more factors are associated with cohesin. Although the residual activity is relatively weak, in one case can rich up to 50% of the wild-type activity (Fig. 4C,D). This is indicative for redundancy mechanisms that are at work *in vivo*, owing to additional cohesin regulators that are absent *in vitro*, such as Pds5A, Pds5B, MCM and chromatin^20,25,29^. Furthermore, we noticed the identical mutations in either MmEsco1 or MmEsco2 evoke Smc3 acetylation deficiencies to a similar qualitative extent (Figs. 4D and 5D). Nonetheless, there are some quantitative differences. This difference between Esco1 and Esco2 might reflect the fact that they act *in vivo* in a very different molecular context and do not have identical binding partners.

Taken together, we propose that two general bases present in Esco enzymes abstract the substrate proton *via* serine residues (Fig. 6). The presence of the catalytic residues D567 and S566 in the CoA binding wall of the active site cleft and E491 and S527 protruding from the wall made of β-strands 5, 6 and 7 suggest that the active sites of Esco1 and Esco2 could be mirror symmetrical. Esco1 and Esco2 sequentially acetylate two distinct, neighboring lysines located in the basic patch of Smc3^37^. An interesting possibility is that the”duplication” of the catalytic residues has evolved as a mechanism required for acetylation of both substrate lysine residues.

## Material and Methods

### MmEsco2^368–592^ cloning, expression and purification

Truncated MmEsco2^368–592^ with a C-terminal His-tag was cloned into the pFL vector^41^. The expression construct was transformed into DH10MultiBacY cells and recombinant bacmid was purified with a QIAprep Spin Miniprep Kit (Qiagen). V_0_ and V_1_ virus generation was performed using Sf9 cells as previously described^42^. Hi5 cells were infected with V_1_ virus for protein expression. Cells were harvested after 48 h and resuspended in lysis buffer (50 mM HEPES pH 7.2, 200 mM NaCl, 10% glycerol, 2 mM DTT, and complete EDTA-free protease inhibitors cocktail [Roche]) and lysed using a microfluidizer. The lysate was cleared by centrifugation and the supernatant was applied onto a 50 ml anion-exchange Q-Sepharose column (GE Healthcare) equilibrated with lysis buffer. The bound proteins were eluted with a linear gradient from 100 mM to 1 M NaCl. The peak fractions containing Esco2 were pooled and applied onto a 1 ml Ni-NTA Superflow column (Qiagen), equilibrated with 20 mM HEPES pH 7.2, 500 mM NaCl, 10% glycerol, 2 mM DTT, and 10 mM imidazole. The bound proteins were eluted with a linear imidazole gradient of 15–250 mM. The peak fractions were pooled, concentrated to a volume of 2 ml and applied onto a S75 16/600 pg size exclusion column (GE Healthcare), equilibrated with 10 mM HEPES pH 7.2, 150 mM KCl, 5% glycerol and 2 mM DTT. Peak fractions were concentrated and stored at −80 °C.

### MmEsco2^368–592^ crystallization and structure determination

MmEsco2^368–592^ was crystallized using the sitting-drop vapor-diffusion method at 20 °C. Crystals were obtained from droplets consisting of 100 nl of MmEsco2^368–592^ (10 mg/ml) and 100 nl crystallization solution (100 mM Tris, 20% (v/v) 2-Methyl-2, 4-pentanediol [MPD], pH 8). After harvesting, crystals were cryoprotected in 15–20% ethylene glycol and flash frozen in liquid nitrogen.

Diffraction data were collected at beamline PXII of SLS (Paul Scherrer Institute, Villigen, Switzerland), processed and scaled using XDS^43^. By making use of the natively bound zinc ion, the crystal structure was determined by single-wavelength anomalous dispersion (SAD) from a 2.3 Å dataset collected at the zinc peak wavelength (Native I in the Supplementary Table S1). The initial model was then refined against a dataset of higher resolution 1.77 Å (Native II in the Supplementary Table S1). The final model was built manually using COOT32^44^ and structure refinement was performed with Phenix33^45^.

### Human ESCO1 cloning, expression and purification

HsESCO1 was cloned into the pFastbac-HTC vector (Invitrogen) with an N-terminal His-tag. HsESCO1 variants was expressed in Sf9 cells. Cells were harvested after 48–72 h and resuspended in lysis buffer (20 mM HEPES pH 7.5, 300 mM NaCl, 10% glycerol, 30 mM imidazole, 1 mM TCEP and complete EDTA-free protease inhibitors cocktail [Roche]) and lysed by sonication. The lysate was cleared by centrifugation. Subsequently, the supernatant was incubated with equilibrated Ni-NTA beads (Qiagen) for 2 h at 4 °C. Bound proteins were washed with high salt buffer (lysis buffer containing 1 M NaCl). Proteins bound to Ni-NTA beads were eluted with lysis buffer containing 150 mM NaCl, 500 mM Imidazole and dialyzed for 16 h against dialysis buffer (20 mM HEPES pH 7.5, 100 mM NaCl, 10% glycerol, 1 mM DTT). The dialyzed samples were snap-frozen.

### Human trimeric and tetrameric cohesin complex cloning, expression and purification

To produce trimer complex, HsSCC1 was cloned into a 438-C vector (Addgene, 55220), containing an N-terminal His-tag followed by a maltose binding protein (MBP) tag and a tobacco etch virus (TEV) protease cleavage site. Cloning into this vector was achieved by the ligation independent cloning (LIC) method. HsSmc3-FLAG and HsSMC1-His in pFastbac and combined SMC1, SMC3-FLAG, SCC1 and His-SA1 in a pFL multibac vector were provided by Jan-Michael Peters lab (Research Institute of Molecular Pathology, Vienna).

The trimer cohesin complex was expressed in Sf9 cells using coinfection with SMC1-His, SMC3-FLAG, and SCC1-MBP viruses. Cells were lysed in lysis buffer (20 mM HEPES pH 7.5, 500 mM NaCl, 10% glycerol, 1 mM DTT and complete EDTA-free protease inhibitors cocktail [Roche]) supplemented with 0.02% NP40 and 1 mM PMSF. After sonication and clarification by centrifugation, the lysate was applied onto a 5 ml amylose column (GE Healthcare) equilibrated with lysis buffer. The bound proteins were eluted with a linear gradient of 10–100 mM maltose. The peak fractions were pooled, concentrated and applied on a S200 16/600 pg size exclusion column (GE Healthcare), equilibrated with 10 mM HEPES pH 7.2, 150 mM KCl, 5% glycerol and 2 mM DTT. Peak fractions were concentrated and snap-frozen.

The tetramer cohesin complex was expressed in Hi5 cells using coinfection with SMC1, SMC3-FLAG, SCC1, and HIS-SA1 viruses. Cells were lysed in lysis buffer (50 mM HEPES pH 7.5, 300 mM NaCl, 10% glycerol, 2 mM DTT, 30 mM imidazole and complete EDTA-free protease inhibitors cocktail [Roche]) supplemented with 1 mM TCEP, 1 mM Pefabloc (Sigma) and 0.05% Tween-20. After sonication and clarification by centrifugation, the supernatant containing tetrameric cohesin was filtered using a 0.8 μm filter (Millipore). Subsequently, the lysate was incubated with 1 ml of Ni-NTA beads for 2 h at 4 °C. Ni-NTA beads were washed with 10 beads volume (BV) of lysis buffer, followed by 10 BV of high salt buffer (lysis buffer containing 1 M NaCl), lysis buffer and finally low salt buffer (lysis buffer containing 150 mM NaCl). Proteins bound to Ni-NTA beads were eluted with lysis buffer containing 150 mM NaCl and 250 mM imidazole. Eluate was incubated with 200 µl anti-FLAGM2 agarose beads (Sigma) for 2 h at 4 °C. The complex was eluted with elution buffer (25 mM HEPES pH 7.5, 150 mM NaCl, 10% glycerol, 1 mM DTT and 0.5 mg ml-1 FLAG peptide) and snap-frozen.

### Site-directed Mutagenesis

Point mutations in HsESCO1, MmEsco1 and MmEsco2 were introduced with the QuikChange II XL site-directed mutagenesis kit (Agilent Technologies) according to the manufacturer’s manual and were verified by DNA sequencing.

### *In vitro* acetylation assay

Acetylation assays were performed by preincubation of 500 nM of trimer (to compare acetylation of tetrameric and trimeric cohesin by HsESCO1, 100 nM we used) or 100 nM of tetramer with 240 µM ATP, 10 µM AcCoA, 3.3 nM pcDNA3.1 plasmid, 25 mM HEPES pH 7.5, 25 mM NaCl, 1 mM MgCl_2_, and 0.05 mg ml^−1^ BSA at 32 °C. After 1 h, 50 nM HsESCO1 and additional NaCl (100 mM final concentration) were added and incubated at 37 °C. The reactions were stopped at the different time points by adding an equal volume of 2X SDS loading buffer, and denatured at 100 °C for 5 min.

### Generation of *Esco1* conditional knockout mouse line

The *Esco1* targeting vector, containing a long (7.3 kb in size) and a short (3.8 kb in size) homology arm, two loxP sites flanking exons 2 and 3 and a FRT-flanked neomycin cassette, was generated by Polygene (Supplementary Fig. S3A). After Validation of the targeting construct, we linearized the vector by NotI restriction endonuclease and electroporated it into the 129/SvPas embryonic stem (ES) cells. Subsequently, neomycin-resistant clones were screened by Southern blotting (Supplementary Fig. S3B). The verified ES cell clones were injected into C57BL/6 J blastocysts. Resulting chimeric animals were mated with ACTB-FLPe mice ubiquitously expressing FLP-recombinase^46^. Removal of the neomycin cassette and germ line transmission were validated by PCR (Supplementary Fig. S3C). To generate *Esco1*^*−/−*^ mice for subsequent MEF isolation, *Esco1*^*fl/fl*^ mice were crossed to a ubiquitous UBC-Cre driver mouse line^47^. For Southern blotting, the probe (361 bp) was amplified with primers TCTCGTCATTTCAGAAACCATC and GCTCACCTATGCTCACATGAAG. For PCR genotyping, a combination of three primers was used (P1: CACCACACTGGCATTAAGTTCTAGG, P2: CCTTACAGTGATGAAACTGAGCACC, P3: GAGTTTCCTGTAGCCAGAGGATGG).

### Cell culture, transfection, synchronization, extract preparation and immunoblotting

Wild-type and indicated mutants of MmEsco1 were cloned into pEF6/Myc-His B vector (Thermo Fisher Scientific) containing C-terminal myc and His tags. Immortalized MEFs^*Esco1−/−*^ cultured in standard medium (DMEM, supplemented with 10% fetal bovine serum [FBS], 100 U/ml penicillin and 100 µg/ml streptomycin) were transiently transfected with the wild-type and mutant versions of *MmEsco1*. To synchronize cells in G1, 36 h after transfection, the medium was changed to DMEM medium supplemented with 10% FBS and 25 µM lovastatin (Thermo Fisher Scientific). Cells were harvested after 24 h and synchronization was assessed by flow cytometry.

Wild-type and mutants *MmEsco2-myc/his* and *H2B-mCherry* were cloned into the pVITRO2-hygro-mcs vector (InvivoGen) in two steps. First, full-length *MmEsco2* was cloned into the pcDNA3.1/myc-His vector (Thermo Fisher Scientific). Subsequently, *MmEsco2-myc/his* and *H2B-mCherry* were amplified from the vectors pcDNA3.1/myc-His and pcDNA3-*H2B-mCherry* (Addgene, 20972), respectively, and cloned into the pVITRO2-hygro-mcs vector. Immortalized MEFs^*Esco2fl/fl*^ were grown to confluence in standard medium and transduced with Ad-Cre-GFP adenoviruses (SignaGen) in low serum medium (DMEM, supplemented with 3% FBS). After two days, the medium was changed to fresh low-serum medium and cells were cultured for another 48 h. Immortalized MEFs^*Esco2−/−*^ were stably transfected with wild-type and mutant versions of Esco2. Clones were then screened using a plate reader by measuring mCherry fluorescence levels. Subsequently, clones that stably expressed the mutant proteins close to the endogenous MmEsco2 level were selected using Western blotting. For synchronization, cells were treated twice with 2 mM thymidine for 14 h with an intermittent release of 9 h. Cells were harvested 2 h after second thymidine release and further processed for subsequent analyses.

For whole‐cell extracts, cells were collected, washed in 1x cold PBS, resuspended in 2X SDS loading buffer and sonicated. Chromatin fractionation was performed according to the protocol described^48^.

### Prometaphase chromosome spreads and immunofluorescence analysis

MEFs were grown in standard culture medium. Cells at 60% confluency were arrested using nocodazole (400 ng ml^−1^) for 4 h. Mitotic cells were harvested by shaking off and incubated in 1 ml of 75 mM KCl for 20 min at 37 °C. Prometaphase chromosomes were fixed in methanol:acetic acid (3:1), dropped onto humidified positively charged microscope slides and visualized using DAPI staining.

### Antibodies

Rabbit antibody against MmEsco1 was generated using a haemocyanin-conjugated peptide comprising amino acids 521 to 606 of mouse Esco1 (1:1000). The following previously described custom-made antibodies were used: anti-Esco2^24^ (1:1000), mouse anti-acetyl-Smc3 (a gift from K. Shirahige)^21^ (1:1000). The following commercial antibodies were used: rabbit anti-Smc3 (Cell Signaling D47B5, 1:3000), HRP-conjugated mouse anti-TBP (Abcam 197874, 1:5000), HRP-conjugated mouse anti-His tag (Novus 31055H, 1:1000).

## Supplementary information

Supplementary information.

## Data Availability

All data generated or analyzed during this study are included in the manuscript and supporting files.
