# Supplementary material for: Alternative catalytic residues in the active site of Esco acetyltransferases
Source: Sci Rep. 2020 Jun 17;10:9828. doi: 10.1038/s41598-020-66795-z (PMC7300003; doi:10.1038/s41598-020-66795-z)
Supplement: Supplementary file 1 — Supplementary information. [file 41598_2020_66795_MOESM1_ESM.pdf]

Supplementary information

Alternative catalytic residues in the active site of Esco acetyltransferases

Tahereh Ajam[1], Inessa De[2,3], Nikolai Petkau[1], Gabriela Whelan[1], Vladimir Pena[2,4], Gregor Eichele[1,5]

[1]Genes and Behavior Department, Max Planck Institute for Biophysical Chemistry, 37077 Göttingen, Germany
[2]Research Group Macromolecular Crystallography, Max Planck Institute for Biophysical Chemistry, 37077 Göttingen, Germany
[3]Current address: European Molecular Biology Laboratory (EMBL), Structural and Computational Biology Unit, Meyerhofstrasse 1, 69117 Heidelberg, Germany
[4]Current address: Structural Biology Division, The Institute of Cancer Research, SW3 6JB London, United Kingdom
[5]Lead contact
Correspondence and requests for materials should be addressed to G.E. (email: gregor.eichele@mpibpc.mpg.de) and V.P. (email: vlad.pena@icr.ac.uk)

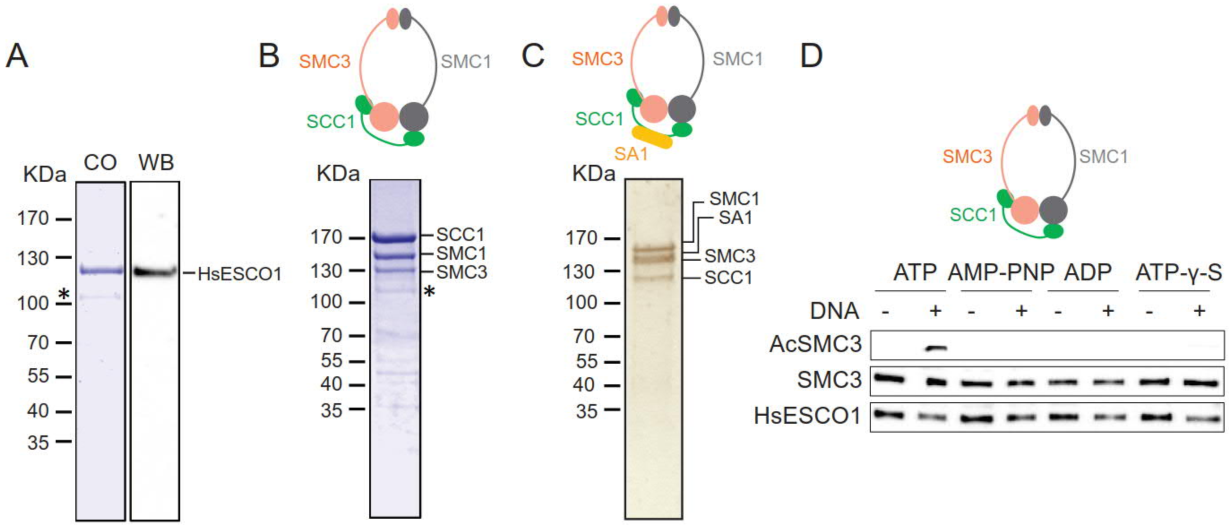

Supplementary Figure S1. Purification and functional characterization of human Esco1 and cohesin complex. (**A**) Purified HsESCO1 analyzed by SDS-PAGE followed by Coomassie blue staining (CO) or Western blotting (WB). The protein was detected as a single protein band with a molecular mass of about 120 kDa. The asterisk indicates a minor contaminant. (**B**) Purified human trimeric cohesin analyzed by SDS-PAGE followed by Coomassie staining. The bands corresponding to SCC1, SMC1 and SMC3 are indicated on the right side. The asterisk indicates a minor contaminant. (**C**) Purified human tetrameric cohesin analyzed by SDS-PAGE followed by silver staining. The bands corresponding to SCC1, SMC1, SMC3 and SA1 are indicated on the right side. (**D**) The trimeric cohesin complex was incubated with HsESCO1 and AcCoA in the presence of ATP, adenylyl-imidodiphosphate (AMP-PNP), adenosine diphosphate (ADP) or adenosine 5′-[γ-thio] triphosphate (ATP-γ-S). The level of SMC3 acetylation level was analyzed by Western blotting. Figure adapted from [50].

A

atom of the residues in MmEsco2	distance between oxygen atom of the catalytic candidate residues and ε-amino group of K105 and K106 in xEco2	
	**K105**	**K106**
**γ-oxygen of S566**	5.0	4.9
**δ-oxygen of D567**	4.4	5.4
**γ-oxygen of S527**	8.7	8.3
**ε-oxygen of E491**	9.8	9.6

B

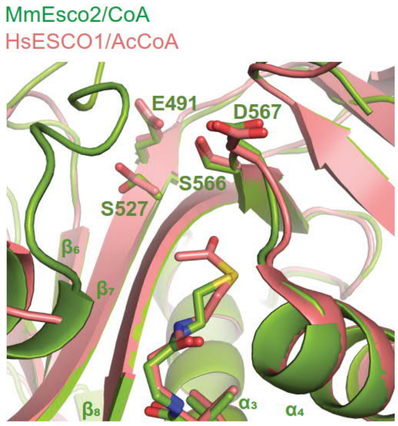

Supplementary Figure S2. Active site of MmEsco2$^{368\text{-}592}$. (**A**) The distance of relevant atoms of the putative catalytic residues to the ε-amino group of K105/K106 in the active site of the MmEsco2$^{368\text{-}592}$/K105-CoA and MmEsco2$^{368\text{-}592}$/K106-CoA models. The distances are shown in angstrom. (**B**) Close-up view of the active sites of MmEsco2/CoA and HsESCO1/AcCoA. Putative catalytic residues of MmEsco2 (green) and HsESCO1 (raspberry; PDB ID code 4MXE) show high structural conservation. Figure S2B adapted from [50].

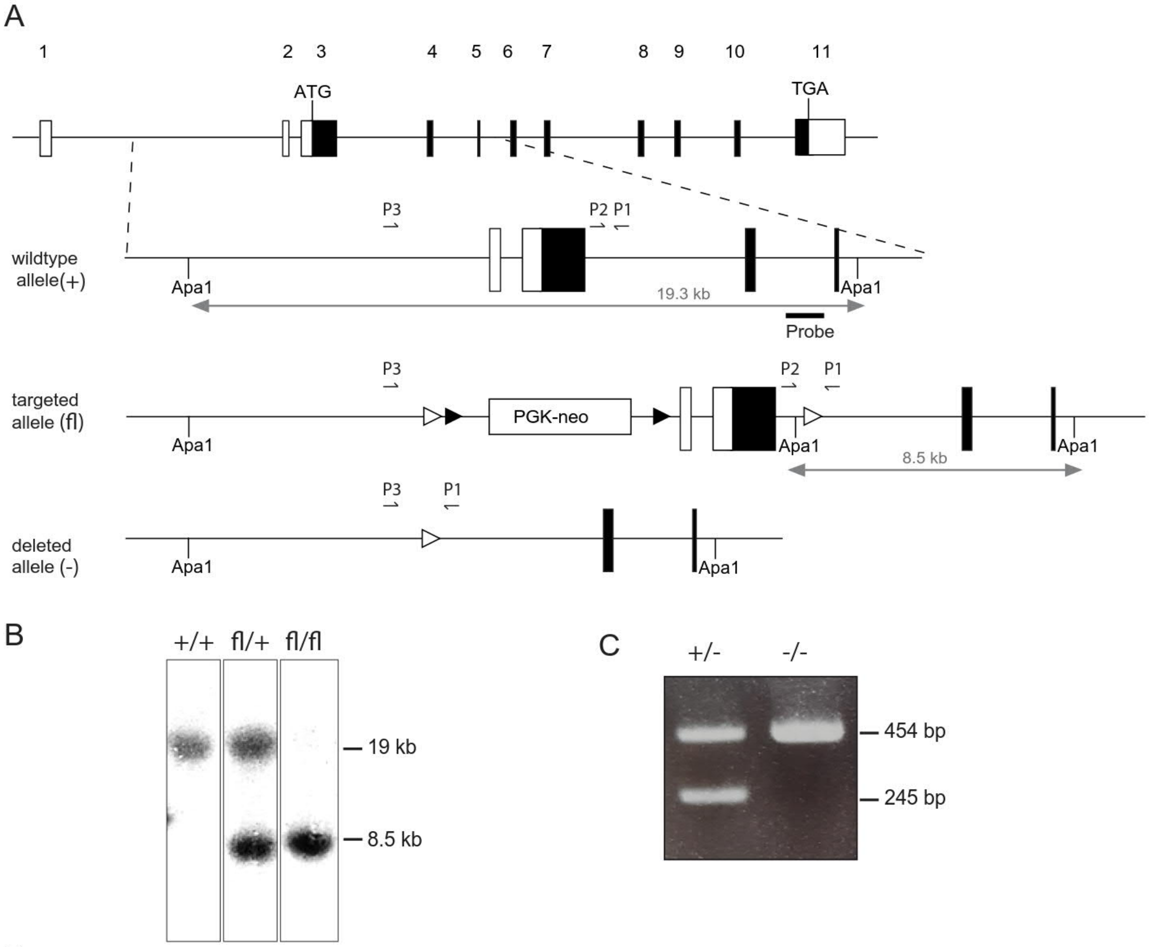

D

Mouse line	Total number of animals	Number of animals (%) homozygous (Δ/Δ)	heterozygous (+/Δ)	wild type (+/+)
Esco1$^{-/-}$	217	34 (16%)	51 (23%)	132 (61%)

Supplementary Figure S3. Generation of Esco1 conditional knockout mouse line. (**A**) Schematic representation of the *Esco1* wild-type locus (+), targeted allele (fl), and the null *Esco1* allele (-) created by Cre-mediated recombination. *LoxP* and *FRT* sites are marked by an empty or black triangle, respectively. (**B**) Southern blot of genomic DNA from mouse tails carrying the alleles indicated, digested with ApaI. The probe used is located between exons 4 and 5, as shown in A. (**C**) PCR genotyping of Esco1 alleles using the three primers indicated in A. The wild-type allele produces a 245-bp amplicon (primers P1, P2). Deletion of the LoxP-flanked region of the Esco1 locus leads to a 454-bp fragment (primers P1, P3). (**D**) Viability statistics of *Esco1*$^{-/-}$ mouse line from heterozygous mating. Mice homozygously deficient for Esco1 are designated as *Esco1*$^{-/-}$. Note that in *Esco1*$^{-/-}$, the homozygous animals were born in a sub-Mendelian ratio, instead of 25 % only 16% of the newborn animals were homozygous.

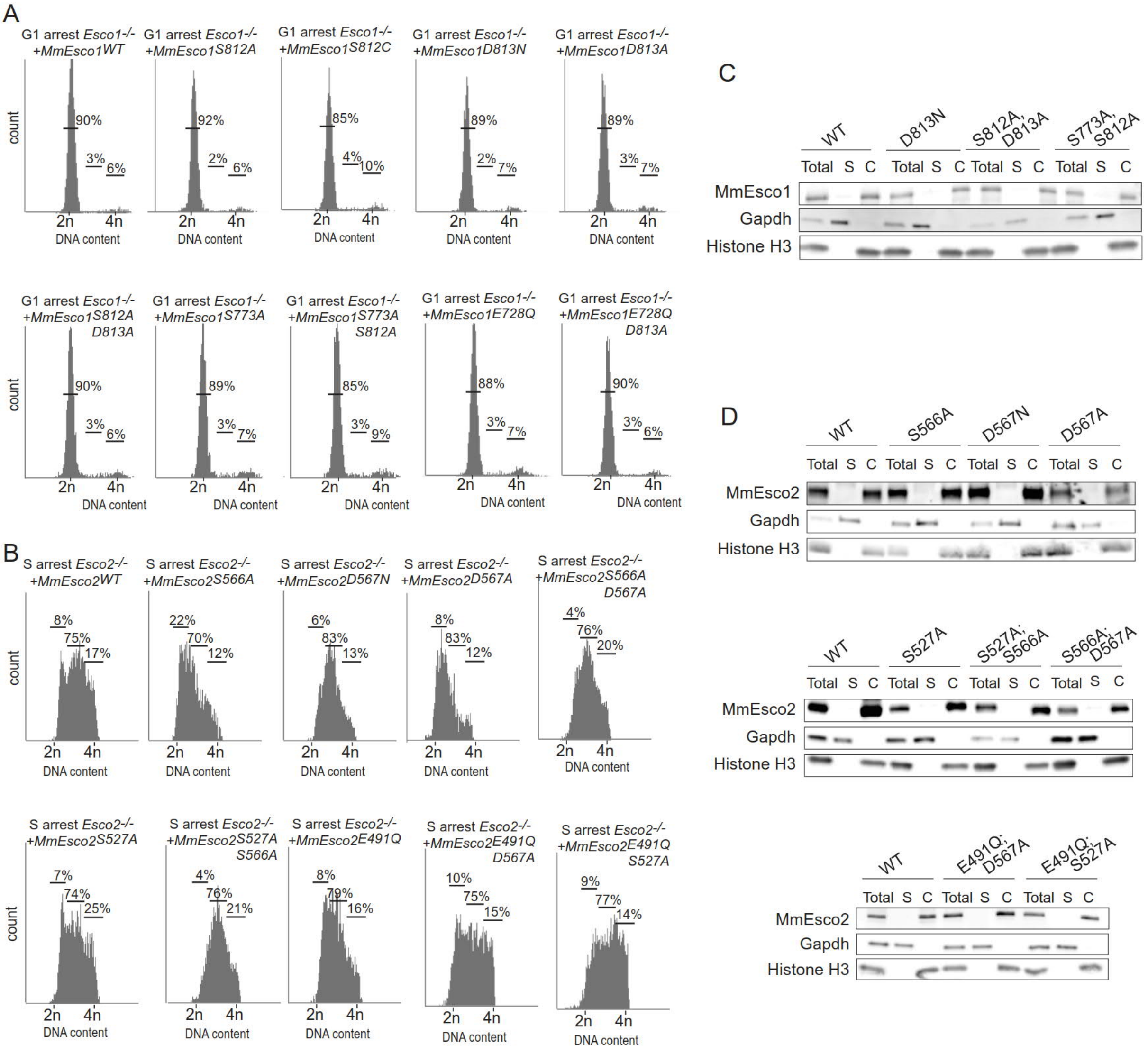

Supplementary Figure S4. *In vivo* characterization of Esco1 and Esco2 mutants. (**A**) Flow cytometry profiles of G1-phase arrested MEFs$^{Esco1-/-}$ expressing wild-type or mutant MmEsco1 (**B**) Flow cytometry profiles of S-phase arrested MEFs$^{Esco2-/-}$ expressing wild-type or mutant MmEsco2. In A and B, the numbers show the percentage of cells in G1, S, G2/M phase. (**C**) Cell fractionation analysis of MEFs$^{Esco1-/-}$ expressing wild-type and catalytically dead mutants of MmEsco1-myc. (**D**) Cell fractionation analysis of MEFs$^{Esco2-/-}$ expressing equivalent amounts of ectopic MmEsco2-myc variants. In C and D, Total: total cell lysate S: soluble fraction, C: chromatin. Figure adapted from [50].

Original unprocessed images of membranes Figure 3

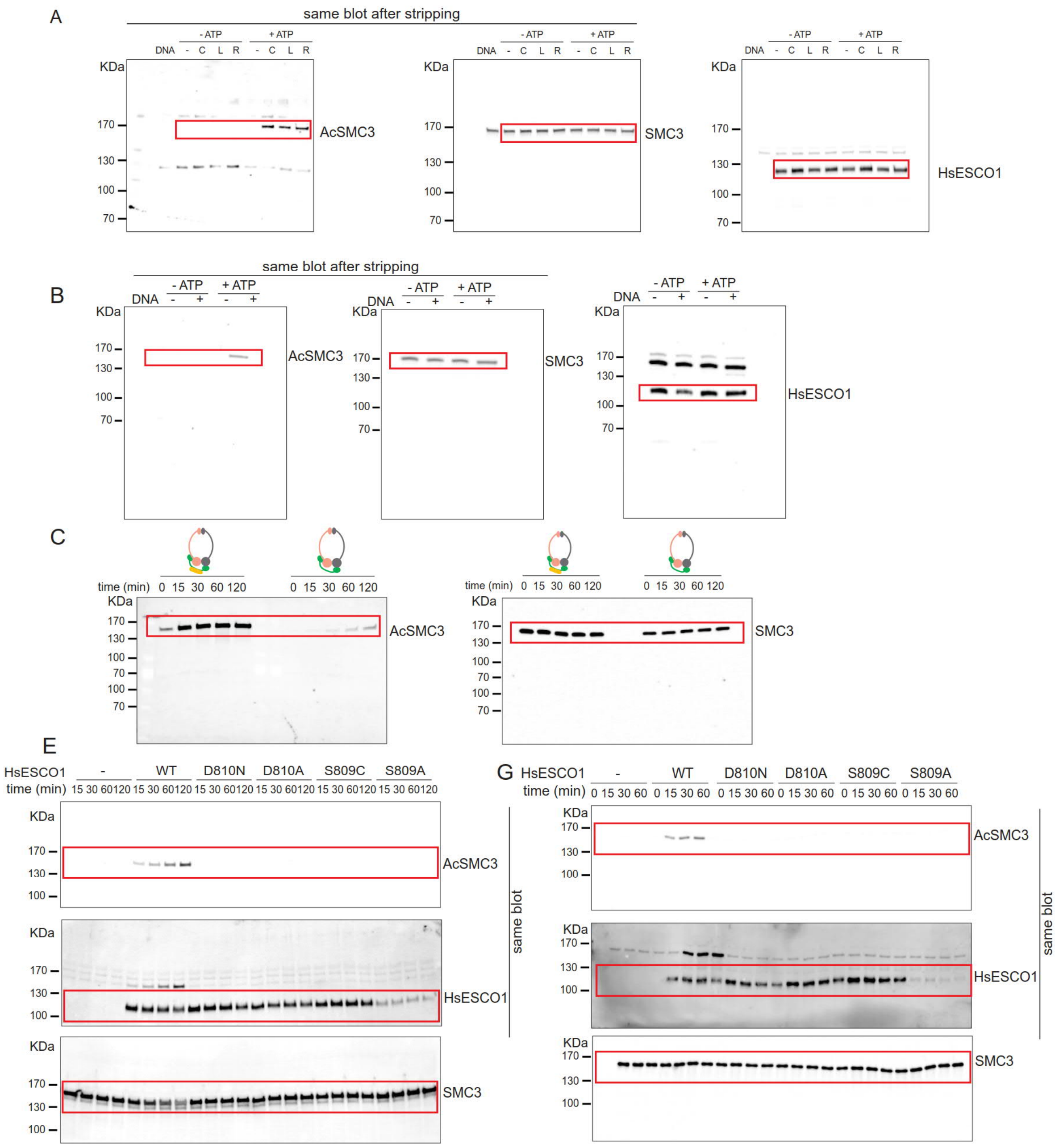

Original unprocessed images of membranes Figure 4B

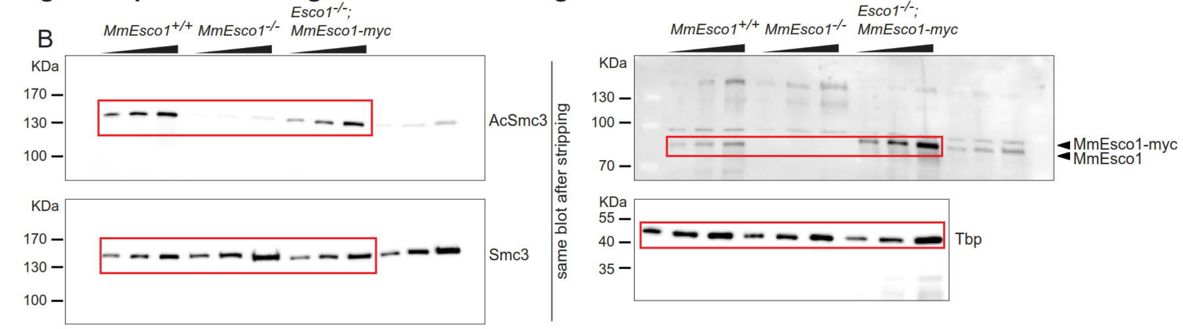

Original unprocessed images of membranes Figure 4C

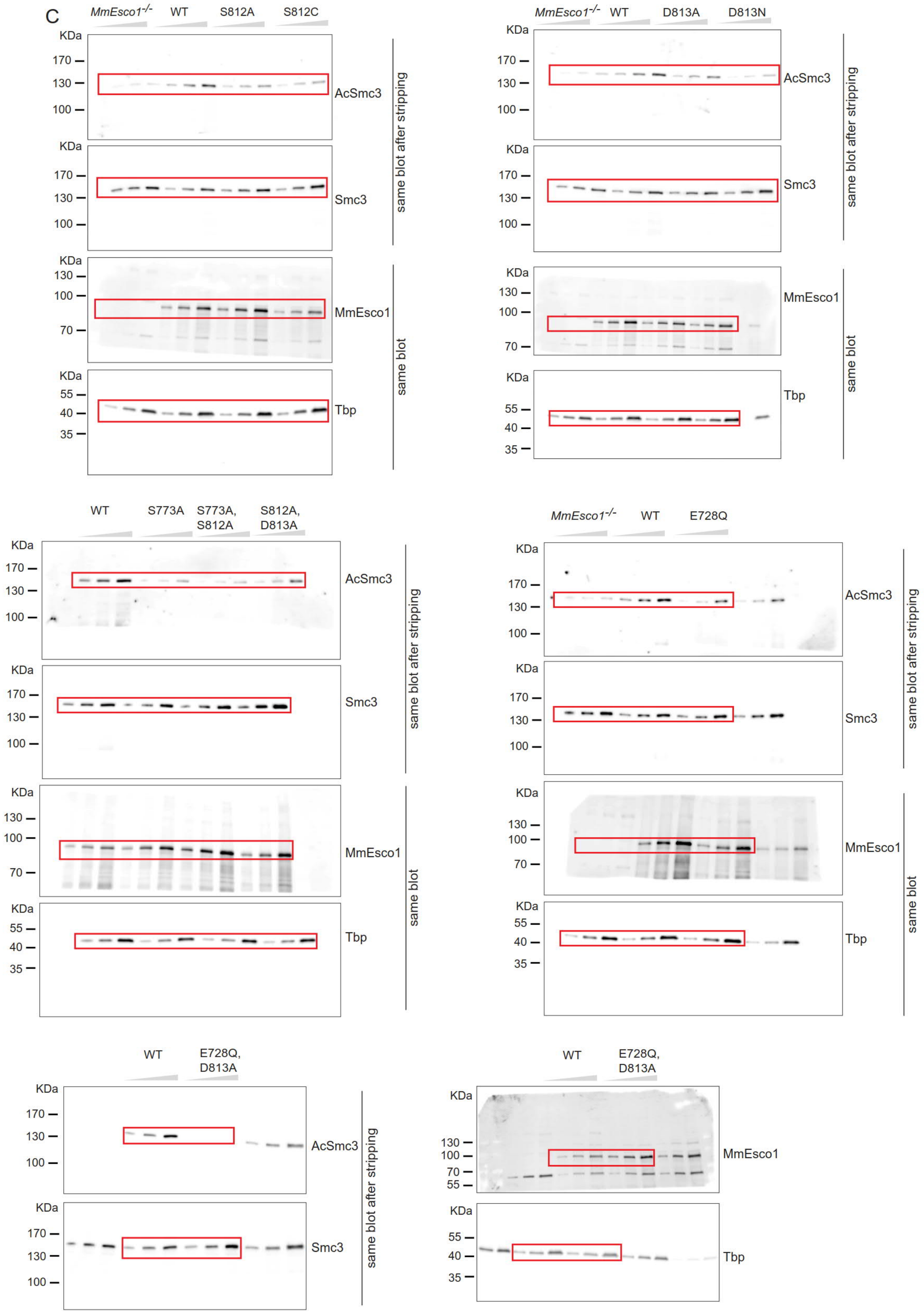

Original unprocessed images of membranes Figure 5

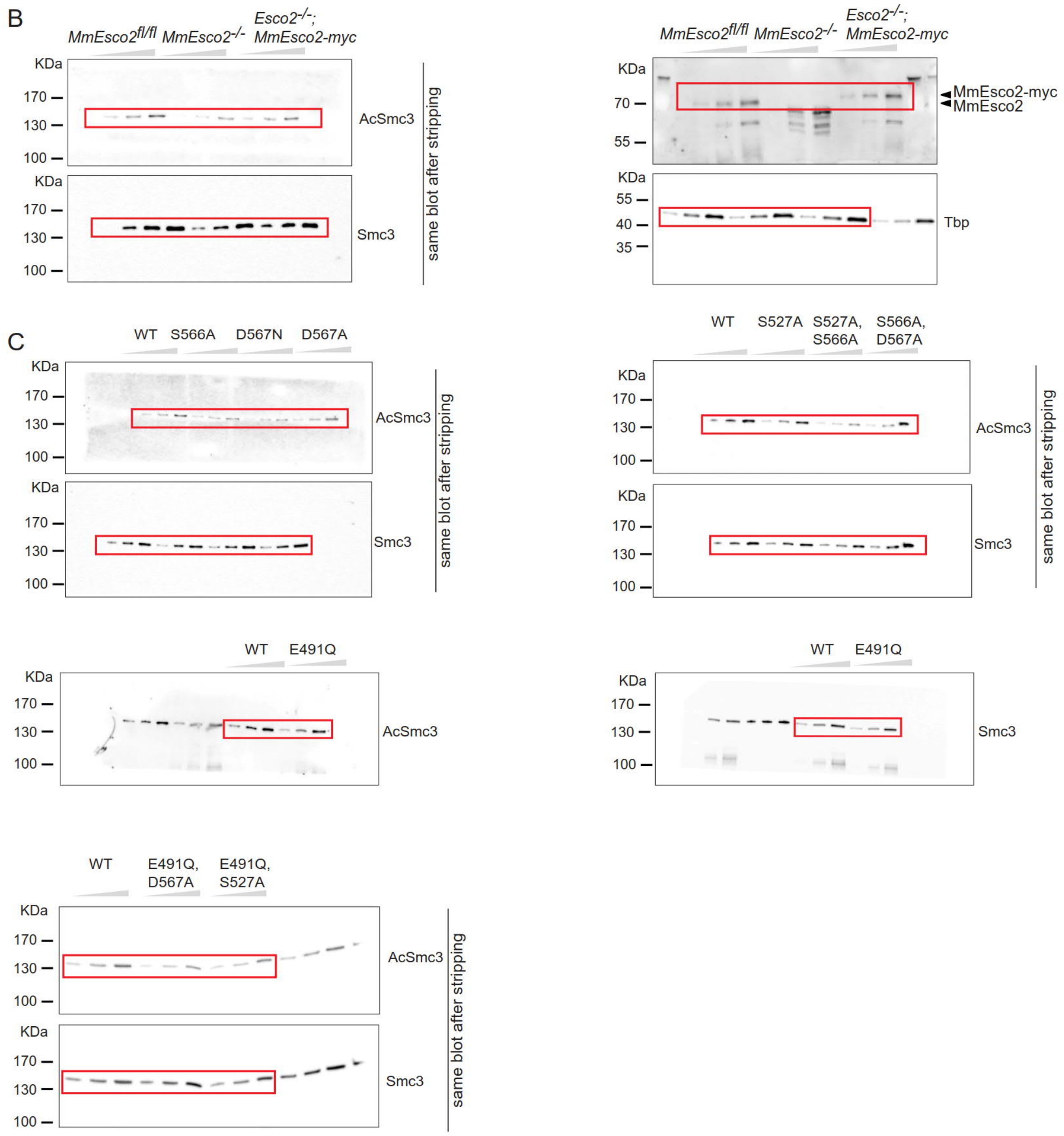

Supplementary Figure S5. Original blots presented in the manuscript. Red rectangles are the parts cropped from original blots. *Same blot after stripping* indicates that the same blot was immunostained with another antibody after stripping the first antibody (Figs. 3A and 3B) (Figs. 4B and 4C) (Figs. 5B and 5C). *Same blot* indicates that after protein transfer from the gel, the blot was cut into two parts for staining with different antibodies (Fig. 4C) or indicates a blot that was restained with another antibody without stripping (Figs. 3E and 3G). Other blots shown were from the same sample volumes loaded on separate gels, blotted separately and stained with different antibody (Figs. 3A, 3B, 3C and 3E) (Figs. 4B and 4C) (Figs. 5B and 5C).

Original unprocessed images of membranes Supplementary Figure S1

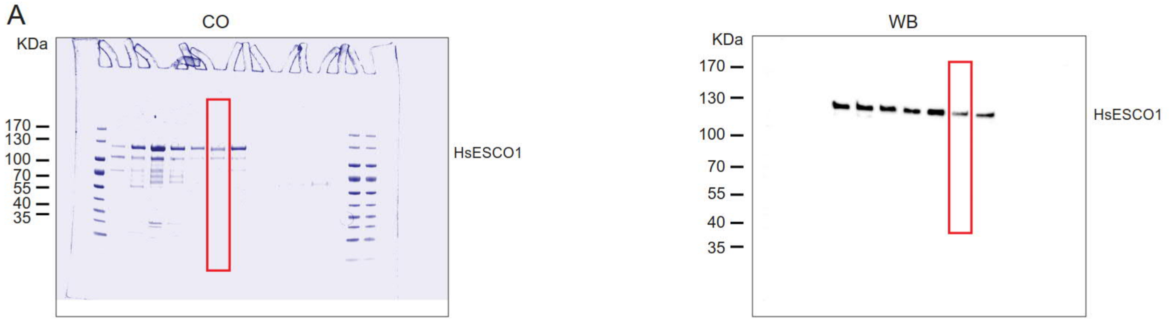

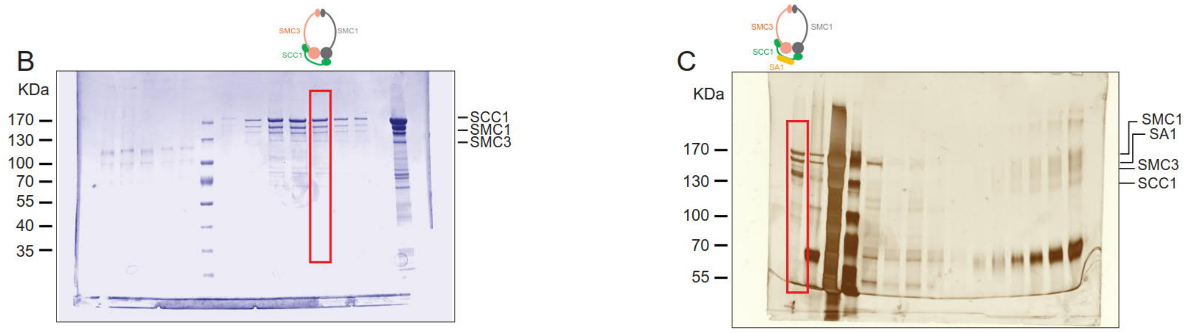

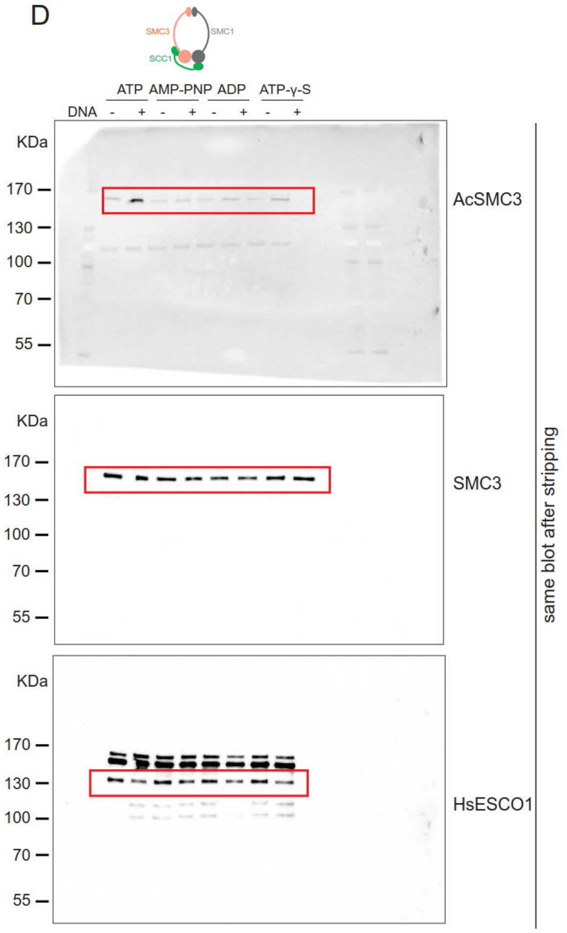

Original unprocessed images of membranes Supplementary Figure S3

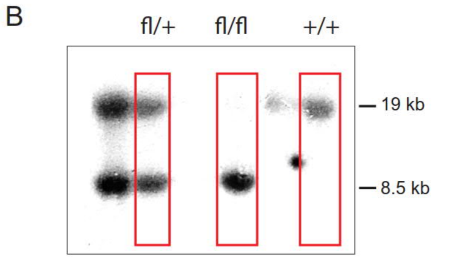

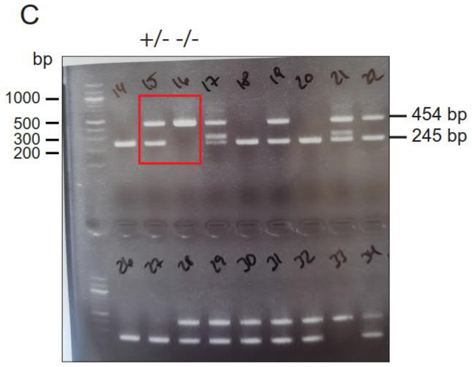

Original unprocessed images of membranes Supplementary Figure S4

C

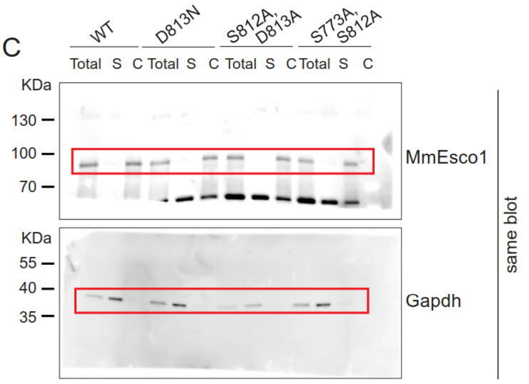

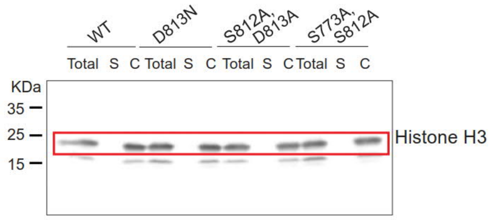

D

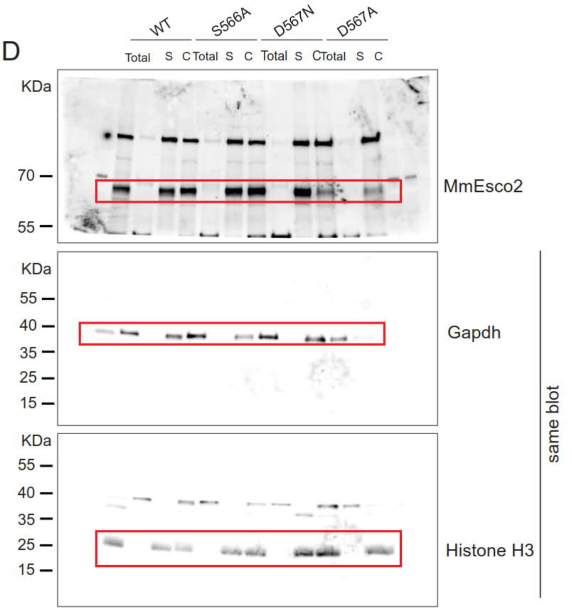

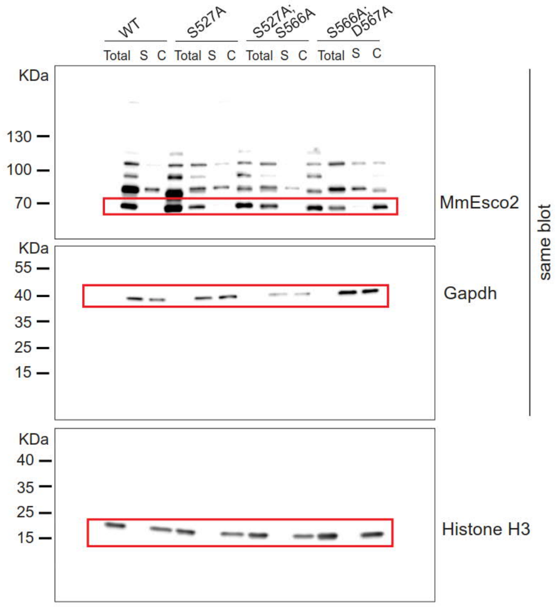

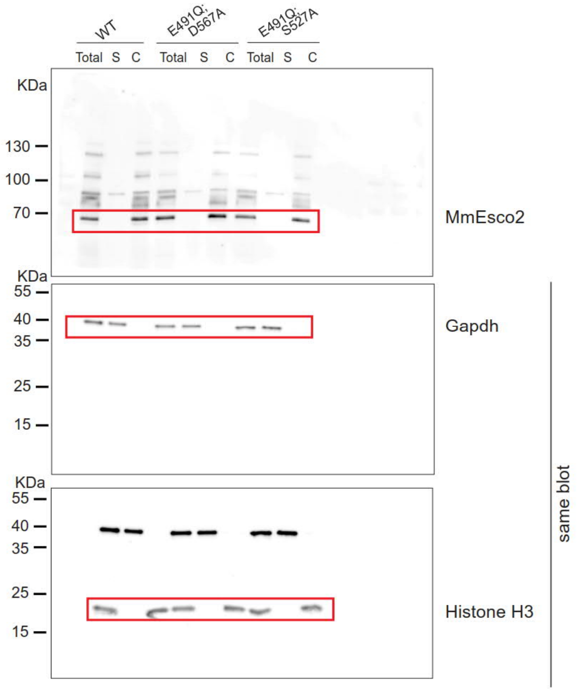

Supplementary Figure S6. Original blots and gels presented in the supplementary information. Red rectangles are the areas cropped from original blots. Same blot after stripping indicates that the same blot was immunostained with another antibody after stripping the first antibody (Fig. S1D). Same blot indicates that after protein transfer from the gel, the blot was cut into two parts for staining with different antibodies (Figs. S4C and S4D) or indicates a blot that was restained with another antibody without stripping (Fig. S4D). Other blots shown were from the same sample volumes loaded on separate gels, blotted separately and stained with different antibody (Figs. S4C and S4D). Fig. S1 presents gels with Coomassie Blue and silver staining. In Fig. S1, the western blot is indicated with WB. Fig. S3B shows the southern blot and Fig. S3C shows the DNA fragments on agarose gel after electrophoresis.

Supplementary Table S1: Data collection, phasing and refinement statistics.

	Native I - Zn K-Edge	Native II
**Data collection**		
Space group	$P4_3$	$P4_3$
Cell dimensions		
a, *b*, *c* (Å)	52.6, 52.6, 106.1	52.7, 52.7, 107.5
α, β, γ (°)	90, 90, 90	90, 90, 90
Wavelength	1.28	1.0
Resolution (Å)	50.0-2.3 (2.3)	47.3-1.77 (1.77)
R_{sym}	2.1 (50.8)	4.5 (46.4)
$I / \sigma I$	22.27 (4.1)	15.2 (4.7)
Completeness (%)	99.6 (99.1)	99.7 (95.8)
Redundancy	7.0 (7.1)	6.7 (6.5)
CC(1/2)	99.9 (93.6)	99.9 (92.8)
**Refinement**		
Resolution (Å)		47.3-1.77
No. reflections		28343
R_{work} / R_{free}		16.9/19.8
No. atoms		
Protein		1606
Ligand/ion		48
Water		158
B-factors		
Protein		37
Ligand/ion		30/37
Water		46.8
R.m.s deviations		
Bond lengths (Å)		0.012
Bond angles (°)		1.236
Ramachandran		
favored (%)		99.0
allowed (%)		1.0
outliers (%)		0

One crystal was used for each dataset. Values in parentheses are for highest-resolution shell.
